# A single-institution retrospective analysis of gastric carcinoma with positive peritoneal lavage cytology and without serosal invasion: A case series

**DOI:** 10.1016/j.amsu.2019.01.003

**Published:** 2019-01-30

**Authors:** Taizo Sakata, Takaomi Takahata, Toshikazu Kimura, Isao Yasuhara, Toru Kojima, Yoshihiro Akazai, Tetsushige Mimura, Alan Kawarai Lefor

**Affiliations:** a Department of Surgery, Okayama Saiseikai General Hospital, Okayama, Japan; b Department of Surgery, Jichi Medical University, Tochigi, Japan

**Keywords:** Gastric carcinoma, Peritoneal cytology, Prognosis, Serosal invasion

## Abstract

**Background and objectives:**

Peritoneal dissemination of gastric cancer is often associated with serosal infiltration. The aim of this study was to evaluate the clinical importance of peritoneal lavage cytology in patients with gastric carcinoma without serosal invasion. The incidence and impact on prognosis of positive cytology were analyzed.

**Methods:**

Of 2768 patients with gastric cancer, outcomes and pathological characteristics of 973 patients were reviewed retrospectively. All patients underwent peritoneal lavage at laparotomy for curative or palliative resection of gastric cancer between 1999 and 2017. Among these, 479 who underwent surgery from January 1999 to March 2012 were also reviewed to analyze 5-year survival.

**Results:**

Of 973 patients enrolled, 338 (35%) did not have serosal invasion, and peritoneal cytology was positive in 4/338 (1.2%). Of these four patients, one had submucosal invasion and three had muscularis propria invasion. Of 635 patients with serosal invasion, peritoneal cytology was positive in 74/635 (12%). Of 479 patients reviewed for survival, cytology was positive in 32/479, with 3/32 (9%) surviving for five years, and cytology was negative in 447 patients with 266/447 (60%) surviving for five years.

**Conclusions:**

Cytologic evaluation should be routinely performed in patients with early-stage gastric cancer.

## Highlights

• Patients with gastric carcinoma without serosal invasion can have positive cytology, although cytologic evaluation has a relatively low sensitivity.
• Positive cytology is associated with a significantly worse prognosis.
• Lymph node metastases and distant metastasis were significant risk factors for positive peritoneal cytology.

## 1 Introduction

Although advances in diagnostic methods and treatment have contributed to decreasing overall mortality from many malignancies, the prognosis of patients with gastric carcinoma is often poor due to a high incidence of peritoneal metastases, which is the most frequent site of recurrence [1]. The 5-year survival rate of patients with positive peritoneal lavage cytology is reported to be just 2% [2]. Several studies showed that peritoneal lavage can identify patients at increased risk of developing peritoneal recurrence [[3], [4], [5]]. In 1998, the Japanese Classification of Gastric Carcinoma published the second English edition based on the 13th Japanese edition that includes the results of cytologic evaluation of peritoneal lavage fluid in the TNM staging system. This identifies patients with positive peritoneal cytology (CY1) as having Stage IV disease. Subsequently, in 2010 the 7th edition of the American Joint Committee on Cancer (AJCC) Staging Manual followed the same classification [6].

Several studies reported that peritoneal dissemination of gastric cancer is often associated with carcinoma infiltrating the serosa [[7], [8], [9]]. While peritoneal metastases are generally assumed to result from the shedding of tumor cells from the serosal surface of the primary tumor, it has been reported that approximately 0.5% of patients with early gastric carcinoma and 5% of patients gastric cancer invading the muscularis propria develop peritoneal recurrence despite undergoing a curative resection [10].

We report one patient with gastric cancer invading the submucosa and three with muscularis propria invasion who presented with positive peritoneal cytology. The aim of this study was to analyze the characteristics of these patients and evaluate the clinical importance of peritoneal lavage cytology for the evaluation of patients with early gastric carcinoma. The sensitivity cytology and impact of positive cytology on prognosis were also analyzed.

## 2 Material and methods

### 2.1 Patient study group

Patients undergoing elective surgery for gastric carcinoma were eligible for this study. Between January 1999 and April 2017, a total of 2768 patients were considered for resection of gastric carcinoma at a large, urban hospital (a general hospital). After excluding recurrent, esophagogastric, non-adenocarcinoma, double cancers, and patients who did not undergo peritoneal lavage with cytologic analysis, we analyzed the data for 973 patients. Throughout study period, board certified gastrointestinal and general surgeons performed surgery. The work has been reported in line with the PROCESS criteria [11].

Between January 1, 1999, and December 31, 2009, a total of 1847 patients with gastric carcinoma underwent resection, and were followed for five years or until death with surveillance according to Japanese gastric cancer treatment guidelines [12]. Of these, 610 patients underwent intraoperative peritoneal lavage. Throughout this manuscript, we use the term cytology analysis to refer to conventional cytologic analysis. To evaluate the effect of positive peritoneal lavage cytology on survival, we retrospectively analyzed 479/610 patients who fulfilled the following criteria: (1) complete macroscopic and microscopic tumor resection (R0) in which the peritoneal cytology status is not taken into consideration, (2) no neo-adjuvant therapy, (3) no other sites of malignancy, and (4) a postoperative survival of at least three months to exclude the effect of postoperative complications on patient survival.

### 2.2 Method of peritoneal lavage and specimen preparation

Immediately after entering the abdominal cavity, 100 ml 0.9% saline was instilled into the left subphrenic area and the pouch of Douglas. A sample of at least 60 ml was aspirated before manipulation of the primary tumor. The sample was centrifuged for 5 min at 1500 rpm. The buffy coat layer containing nucleated cells was placed onto a glass slide and fixed with 95% ethanol. The slide was stained by Papanicolaou staining and read by a cytotechnologist and an experienced pathologist. Cytological findings were evaluated based on Papanicolaou's classification. Class IV and V were defined as positive, denoted as CY1.

### 2.3 Statistical analysis

The chi-squared test was used for group comparisons. Survival rates were calculated by Kaplan-Meier analysis and differences between groups were analyzed using the log-rank test. Cox regression analysis was used for multivariate analysis. A p-value less than 0.05 was considered statistically significant. All statistical analyses were performed with EZR (Saitama Medical Center, Jichi Medical University, Saitama, Japan), which is a graphical user interface for R (The R Foundation for Statistical Computing, Vienna, Austria) [13].

## 3 Results

Of 973 patients who met the inclusion criteria and had peritoneal lavage cytology evaluation, 635/973 (65%) had tumor invading the serosa and 74/635 (12%) had positive peritoneal cytology. There were 338/973 (35%) without serosal invasion, and 4/338 (1.2%) had positive peritoneal cytology. The clinicopathological findings of these four patients with gastric carcinoma patients without serosal invasion and with positive peritoneal cytology are summarized in Table 1.

**Table 1:** Clinicopathological findings in four patients with gastric carcinoma without serosal invasion and positive peritoneal lavage cytology.

Patient	1	2	3		4
Age (years)	25	63	69		83

Gender	Male	Male	Male		Male

Tumor Size (mm)	40 × 35	45 × 75	① 23 × 28	② 12 × 12	65 × 55

Macroscopic tumor type	pType 0-IIc	pType IIc + IIa	pType 3	pType IIc-like	pType 5

Depth of tumor invasion (T)	MP	MP	MP	MP	SM2

Lymph node metastases (N)	N1 (2/46)	N3 (21/67)	N0 (0/61)		N3 (16/53)

Metastases (other than positive cytology)	–	–	–	–	–

Distant metastases	–	16a2 (1/1)	–	–	–

Histologic type	Undifferentiated	Undifferentiated	Differentiated		Undifferentiated
	por	por	tub2	tub1	por
Cancer stromal volume	schirrous	intermediate	intermediate	intermediate	intermediate

Infiltrative pattern	INFγ	INFγ	INFβ	INFβ	INFβ

Lymphatic invasion (ly)	ly1	ly3	ly1	ly0	ly3

Venous invasion (v)	v1	v1	v0	v0	v2

HER2 status	n/a	positive	negative	n/a	n/a

Other findings				Ulcer scar	Lymphatic invasion in gallbladder (M1)

SM: Submucosa MP: Muscularis propria.

### 3.1 Histopathological features

Correlation between the results of cytology evaluation and histopathological features in patients undergoing curative resection is shown in Table 2. Peritoneal lavage fluid cytology was positive in a total of 78/973 (8.0%) of patients. One patient had an early gastric carcinoma (invasion no deeper than the submucosa), and three patients had tumor invading the muscularis propria, with positive peritoneal cytology.

**Table 2:** Correlation cytology results and other factors in 973 patients undergoing gastric resection.

All Patients						
Factor	(n = 973)			Conventional cytology findings		
	*n*		(%)	Negative *n*	Positive *n*	Positive (%)
Gender						
Male	639		66%	591	48	
Female	334		34%	304	30	
Depth of tumor invasion (T)						
T1a(mm)	84		9%	84	0	0%
T1b(sm)	136		14%	135	1	0.7%
T2(mp)	118		12%	115	3	2.5%
T3(ss)	124		13%	122	2	1.6%
T4(se)	454		47%	393	61	13%
T4b(si)	57		6%	46	11	19%
Lymph node metastases (N)						
N0	410		42%	404	6	1.5%
N1	139		14%	132	7	5.0%
N2	148		15%	138	10	6.8%
N3a	152		16%	131	21	14%
N3b	121		12%	87	34	28%
Metastases (other than positive cytology)						
0	783		81%	757	26	3.3%
1	187		19%	135	52	28%
Distant metastases (M)						
0	948		97%	875	73	7.7%
1	25		3%	20	5	20%
Histologic type						
*Differentiated*						
papillary	21		2%	21	0	0%
well differentiated	135		10%	132	3	2.2%
moderately differentiated	195		15%	178	17	8.7%
total	351					5.7%
*Undifferentiated*						
poorly differentiated	566		43%	511	55	9.7%
signet ring cell	28		2%	27	1	3.6%
mucinous	28		2%	26	2	7.1%
total		622				9.3%
Cancer stromal volume						
med	157		18%	151	6	3.8%
sci	252		29%	209	43	17%
int	467		53%	440	27	5.8%
Infiltrative pattern						
a	79		8%	79	0	0%
b	566		59%	545	21	3.7%
c	319		33%	263	56	18%
Lymphatic invasion (ly)						
0	206		21%	205	1	0.5%
1	453		47%	431	22	4.9%
2	228		24%	198	30	13%
3	83		9%	60	23	28%
Venous invasion (v)						
0	335		35%	329	6	1.8%
1	437		45%	394	43	9.8%
2	169		17%	144	25	15%
3	29		3%	27	2	6.9%

When reviewing the data for all patients (N = 2768) who underwent resection of gastric carcinoma, we noticed that peritoneal lavage was performed more commonly in patients with advanced tumors, with a tendency to omit cytology evaluation in patients with less invasive tumors. Most patients who underwent laparoscopic resection did not undergo peritoneal lavage since laparoscopic resection is usually performed in patients with early-stage gastric carcinoma.

### 3.2 Multivariate analysis of the factors associated with positive cytology

In univariate analysis, depth of tumor invasion, presence of lymph node metastases, metastases to other organs, histologic type, and capillary invasion correlated significantly with positive peritoneal cytology. Multivariate analysis showed that lymph node metastases and distant metastasis were significant risk factors for positive peritoneal cytology (Table 3).

**Table 3:** Multivariable analysis.

Variable^a^	Category	Univariable analysis	Multivariable analysis		Confidence interval
		*P*-value	*P*-value	Odds ratio	
Depth of tumor invasion (pT)	T1,2/T3,4	<0.0001	n.s.	–	–
Lymph nodes metastases (pN)	N0/N1,2,3a,3b	<0.0001	0.0002	5.3	2.2–12.6
Metastases (other than positive cytology)	M0/M1	<0.0001	<0.0001	7.9	4.7–13.4
Distant metastases (M)	Yes/no	0.0433	n.s.	–	–
Histologic type	Differentiated/Undifferentiated	0.0492	n.s.	–	–
Lymphatic invasion (ly)	Yes/no	<0.0001	n.s.	–	–
Venous invasion (v)	Yes/no	<0.0001	n.s.	–	–

a Variables selected based on significance in univariate analysis.

### 3.3 Case reports of the patients who showed positive cytology without serosal invasion by tumor

In order to highlight the clinicopathologic characteristics of the four patients without serosal invasion by tumor, but with positive peritoneal cytology, we review each of them here briefly.

Patient 1: A 25-year-old male underwent total gastrectomy including a D3 lymph node dissection for advanced gastric carcinoma, preoperatively staged as T3 (SS), N0, M0, cStage II. Peritoneal lavage showed poorly differentiated adenocarcinoma (Class V). Pathological diagnosis showed the depth of invasion was T2 (invading the muscularis propria). He received adjuvant chemotherapy, biweekly paclitaxel for one year and oral administration of S-1 (TS-1; tegafur, gimeracil, oteracil potassium). At 13 years after resection, there is no evidence of recurrence.

Patient 2: A 63-year-old male underwent distal gastrectomy with D2 lymph node dissection for a type 0-IIc + IIa tumor, preoperatively staged as T1b(SM), N0, M0, cStage IA. Pathological diagnosis showed the depth of invasion was T2 (invading the muscularis propria). Although he began receiving adjuvant chemotherapy, a recurrence was seen on computed tomography scan in the mediastinal and para-aortic areas four months postoperatively. He died 21 months later.

Patient 3: A 69-year-old male underwent total gastrectomy with D2 lymph node dissection for two separate primary gastric carcinomas. Both tumors invaded the muscularis propria (T2) and had lymphatic invasion. He has received adjuvant chemotherapy with docetaxel/S-1 and is free of recurrence at 14 months postoperatively.

Patient 4: An 83-year-old male underwent distal gastrectomy with D2 lymph node dissection for a type III tumor, preoperatively staged as T3(SS), N1, M0, cStage IIb. Pathology examination showed the depth of tumor invasion as T1b(SM2) with significant lymphatic and vascular invasions. There were tumor cells in the lymphatics of the gallbladder, which was incidentally resected for cholelithiasis. He is receiving S-1 monotherapy.

### 3.4 Survival analysis

**Fig. 1:**
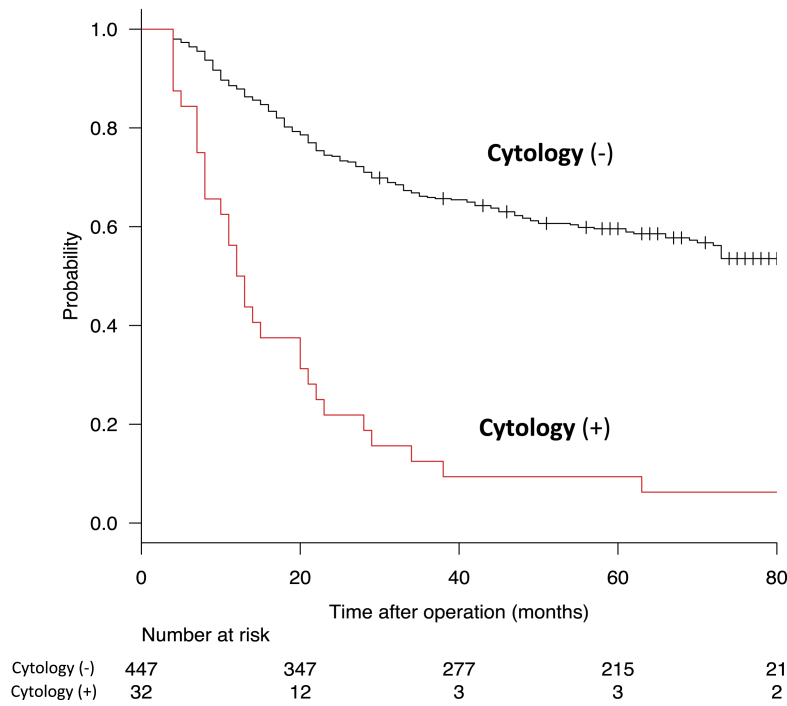
Kaplan–Meier survival curves comparing positive and negative peritoneal cytology in 479 patients with gastric carcinoma.

Of the total group, there were 479 patients treated between January 1999 and December 2009 and had five-year follow-up, comprising the group for survival analysis. Among these patients, positive peritoneal cytology is associated with a significantly worse prognosis (Fig. 1). The median follow-up time was 45 (range 4–141) months. Out of 479, 57 (11.9%) were lost to follow-up due to moving to other places or being unreachable. There were 32/479 patients with positive peritoneal cytology, 3/32 (9.4%) survived for five years. There were 447/479 patients with negative peritoneal cytology, and 266/447 (60%) survived for five years (P < 0.001). The median survival of patients with positive peritoneal cytology was 17.6 months. Fifteen (47%) of the 32 patients with positive peritoneal cytology developed peritoneal recurrences. Distant metastases were found in 10 patients (31%) in addition to peritoneal carcinomatosis.

## 4 Discussion

We identified four patients with gastric carcinoma that did not invade the serosa but had positive peritoneal cytology. The mechanism of how these tumor cells enter the peritoneum has not been elucidated. In existing literature, possible metastatic mechanisms include direct shedding from the serosal surface, followed by invasion of the subperitoneal connective tissue [14]. Other studies suggest that metastases occur through lymphatic vessels, which are located in the submucosal layer [15]. Tumor cells may spread through lymphoreticular organs, called milky spots, which exist throughout the greater omentum [16]. The lymphatic route may explain how early stage gastric cancer, such as T1b tumors, are associated with positive cytology. Although lymphatic invasion was commonly observed in the patients we report, a multivariate analysis failed to show this as a significant risk factor. Bonenkamp et al. reported that risk factors for positive peritoneal cytology include serosal invasion, increasing T stage and lymph node positive disease [17]. Since we believe that submucosal lymphatic vessels are a possible route of tumor spread into the peritoneal cavity, similar cases may be identified if peritoneal cytology is routinely evaluated in patient with gastric cancer.

This analysis identified 4/338 patients (1.2%) with gastric cancer and without serosal invasion with positive peritoneal cytology, compared to 74/635 patients (12%) with gastric cancer who had positive peritoneal cytology and also had serosal invasion by tumor. This result is remarkable for detecting positive peritoneal cytology in patients with gastric carcinoma and without serosal involvement, contrary to previous reports. Previously published data reported increasing T stage, at least T3 or higher, is a significant risk factor for positive peritoneal cytology when conventional cytology analysis was used [[2], [3], [4], [5],^16^]. Despite an excellent specificity, reported to be approximately 100%, it is apparent that cytologic evaluation using Papanicolaou or other classical staining methods has a relatively low sensitivity (11.1%–80%) [18]. Although current guidelines are restricted to cytologic evaluation without providing further information on the technique used, many methods have been investigated to identify the optimal method of tumor cell detection (e.g. immunoassay, immunohistochemistry, and reverse transcriptase-polymerase chain reaction) [19]. This study reported a positive detection rate by cytology of 14% and 20% for submucosa and muscularis propria-invasive gastric carcinoma, respectively, using immunocytochemical staining with the monoclonal anti-epithelial non-cytokeratin antibody Ber-Ep4 [20]. Although the Japanese national health insurance system reimburses for molecular biology assays, most community hospitals still use routine cytology. At present, the use of genetic diagnosis is limited to University hospitals and large cancer centers. It is noteworthy that conventional cytology can detect tumor cells in patients with early-stage gastric carcinoma. A relatively large sample size and a low detection rate may explain these results.

In the present analysis, the 5-year survival rate of patients with positive cytology was 9.3% with a median survival time of 17.6 months. In 1999, Bando et al. reported a 5-year survival rate of 2% and median survival of approximately 12 months [2]. These data included patients treated with surgery alone. Of 32 patients with positive peritoneal cytology, 23 patients (72%) received postoperative chemotherapy, which may have contributed to prolonged survival. The study showed that recent advances in chemotherapy led to a better prognosis for patients with positive peritoneal cytology. In 2012, Kodera et al. reported (the CCOG0301 trial) that patients with positive peritoneal cytology treated by standard gastrectomy followed by S-1 monotherapy until disease progression. The median recurrence-free and overall survival time were 376 and 705 days, and 5-year recurrence-free and overall survival rates were 21 and 26%, respectively [21]. The author suggested that radical surgery could be recommended for patients with positive peritoneal cytology as the sole factor associated with incurability, provided that adequate chemotherapy is given perioperatively.

There is no consensus regarding routinely performing peritoneal cytology into the algorithm of gastric cancer evaluation and treatment. Although the majority of guidelines classify positive peritoneal cytology as metastatic disease, there is no standardization of sampling time and sampling/detection methodology. While the NCCN guidelines recommend staging laparoscopy with peritoneal washings to evaluate peritoneal cytology for patients with stage IB and higher, the European Society for Medical Oncology (ESMO) suggests that this is optional [22,23]. In our institution, although peritoneal washing samples are usually collected intraoperatively, the possibility of detecting tumor cells in patients without serosal invasion has not been well recognized. There has been a tendency not to evaluate cytology during laparoscopic resections, mostly due to the fact that laparoscopic resections are generally performed in patients with early-stage gastric carcinoma. These may have introduced some selection bias among the early stage patients. Further studies are needed with the results of conventional cytology during laparoscopic resection.

In one systematic review, Kwee Rm et al. reported the diagnostic accuracy of overall T staging for endoscopic ultrasound, multi-detector computed tomography scan, and magnetic resonance imaging scans varied between 65% and 92.1%, 77.1% and 88.9%, and 71.4% and 82.6%, respectively [24]. Since the preoperative accuracy of T-staging has limitations, these data highlight the importance of peritoneal lavage cytology, even with conventional cytologic analysis, for patients with stage IB and higher gastric cancer regardless of the preoperative T-stage.

## 5 Conclusion

Peritoneal lavage cytology with conventional analysis is easy and safe to perform at a reasonably low cost. It should be considered to be added to the clinical staging of patients with gastric cancer, since some patients without serosal invasion will have positive peritoneal cytology. Patients with positive peritoneal cytology have a poor prognosis but may have improved survival if they are treated with postoperative chemotherapy.

## Provenance and peer review

Not commissioned, externally peer reviewed.

## Ethical approval

Not applicable.

## Author contribution

Taizo Sakata: design, analysis and writing of paper.

Takaomi Takahata: design and data collection.

Toshikazu Kimura: data collection.

Isao Yasuhara: data collection.

Toru Kojima: reviewed the manuscript.

Yoshihiro Akazai: reviewed the manuscript.

Tetsushige Mimura: reviewed the manuscript.

Alan Kawarai Lefor: reviewed and edited the manuscript.

## Conflicts of interest

There is no conflict of interest to be declared.

## Trial registry number

Researchregistry4392.

## Guarantor

Taizo Sakata.

## Sources of funding

No source to be stated.
